# 16S rRNA gene amplicon sequencing of gut microbiota in gestational diabetes mellitus and their correlation with disease risk factors

**DOI:** 10.1007/s40618-021-01595-4

**Published:** 2021-07-24

**Authors:** J. Wei, Y. Qing, H. Zhou, J. Liu, C. Qi, J. Gao

**Affiliations:** 1 Department of Obstetrics, The Affiliated Changzhou No.2 People’s Hospital of Nanjing Medical University, Changzhou, 213003 China; 2 Bengbu Medical College, Bengbu, China; 3 Dalian Medical University, Dalian, China; 4 Diabetes Mellitus Research Institute of Changzhou, Changzhou, China; 5 Medical Research Center, The Affiliated Changzhou No.2 People’s Hospital of Nanjing Medical University, Changzhou, China; 6 Department of Endocrinology and Metabolism, The Affiliated Changzhou No.2 People’s Hospital of Nanjing Medical University, Changzhou, 213003 China

**Keywords:** Gestational diabetes mellitus, Gut microbiota, 16S rRNA gene amplicon sequencing, qPCR

## Abstract

**Purpose:**

Although the gut microbiota (GM) are associated with various diseases, their role in gestational diabetes mellitus (GDM) remains uncharacterized. Further study is urgently needed to expose the real relationship between GM and GDM.

**Methods:**

We performed a prospective study in 33 pregnant Chinese individuals [15, GDM; 18, normal glucose tolerance (NGT)] to observe the fecal microbiota by 16S rRNA gene amplicon sequencing at 24–28 weeks of gestational age after a standard 75 g oral glucose tolerance test. Linear regression analysis was employed to assess the relationships between the GM and GDM clinical parameters.

**Results:**

Sequencing showed no difference in the microbiota alpha diversity but a significant difference in the beta diversity between the GDM and NGT groups, with the relative abundances of *Ruminococcus bromii*, *Clostridium colinum*, and *Streptococcus infantis* being higher in the GDM group (*P* < 0.05). The quantitative PCR results validated the putative bacterial markers of *R. bromii* and *S. infantis*. Moreover, a strong positive correlation was found between *S. infantis* and blood glucose levels after adjusting for body mass index (*P* < 0.05).

**Conclusion:**

Three abnormally expressed intestinal bacteria (*R. bromii*, *C. colinum,* and *S. infantis*) were identified in GDM patients. *S. infantis* may confer an increased risk of GDM. Hence, the GM may serve as a potential therapeutic target for GDM.

**Supplementary Information:**

The online version contains supplementary material available at 10.1007/s40618-021-01595-4.

## Introduction

The prevalence of metabolic diseases during pregnancy has increased globally [1–3], including gestational diabetes mellitus (GDM), which is diagnosed during pregnancy in women with normal glucose metabolism or potentially diminished glucose tolerance before pregnancy [4]. GDM is associated with many adverse maternal and neonatal outcomes [5], such as preeclampsia, cesarean delivery, fetal macrosomia, shoulder dystocia, and neonatal hypoglycemia [6]. In addition, increasing evidence has indicated that GDM is associated with a higher risk of type 2 diabetes mellitus (T2DM) after pregnancy [7]. Although GDM is a transient condition and glucose metabolism often normalizes shortly after delivery, women with GDM have a 40% higher risk of developing T2DM within a 15-year period [8]. Due to its short- and long-term adverse outcomes, GDM is a public health concern [9], and new treatment methods are urgently required [10].

GDM is a disease characterized by abnormal glucose metabolism with a very similar pathogenesis to T2DM [11]. In recent years, the relationship between T2DM pathogenesis and changes in GM has attracted increasing attention, with studies reporting that GM can participate in glucose and fat metabolism and inflammatory and immune responses [12–14] and promote insulin resistance by altering fat absorption and metabolism [15].

Studies have shown that GM can promote GDM development [16]. During normal pregnancy, GM composition has been reported to remain relatively stable [17] or to change dramatically, with a decline in butyrate-producing bacteria, a reduction in alpha diversity, and an increase in beta diversity [18]. Kuang et al. found that the abundance of GM is similar at the phylum and class levels between GDM and control patients [19]. Crusell et al. found that the abundance of GM in GDM patients is aberrant at multiple levels, including phylum and genus levels, compared with the healthy controls [20]. GDM pathogenesis may be due to an increase in placental insulin antagonist hormones (human placental lactogen, estrogen, progesterone, and tumor necrosis factor [21]) during pregnancy, which causes inappropriate insulin secretion by the pancreas [22]. Koren et al. found that the third trimester microbiota induced greater adiposity and insulin insensitivity compared to the first trimesters when different trimester stool was transferred to germ-free mice [18]. The reduced insulin sensitivity of late pregnancy is beneficial for fetal growth and nutrient absorption [18]. Subsequent increases in insulin levels may then cause a compensatory increase in insulin resistance in peripheral tissues [22]. Moreover, women with GDM have a greater reduction in insulin sensitivity and an increase in insulin resistance, and their insulin secretion is not sufficient to maintain euglycemia, which leads to glucose intolerance and GDM [23]. Although GM is thought to be associated with metabolic-related diseases [24–27], its role in GDM remains unclear.

To obtain a comprehensive understanding of the relationship between GM and GDM, we explored the GM composition and abundance in 33 samples [15 from women diagnosed with GDM and 18 from pregnant women with normal glucose tolerance (NGT)] by 16S rRNA gene amplicon sequencing using an Illumina HiSeq (PE 250) platform. Putative bacterial markers were verified by qPCR, and correlations between dominant intestinal bacteria and clinical parameters were identified by linear regression analysis.

## Materials and methods

### Study population and characteristics

A total of 46 sets of fecal samples, blood samples, and medical records were collected from 19 pregnant women with GDM and 27 pregnant women with NGT (control group) from February 2018 to May 2019 at the obstetrics and endocrinology departments of the Affiliated Changzhou No.2 People’s Hospital of Nanjing Medical University. All pregnant women underwent a 75 g oral glucose tolerance test (OGTT) at 24–28 weeks of gestation according to the International Association of Diabetes and Pregnancy Study Group (IADPSG)/World Health Organization (WHO) 2013 criteria (fasting venous plasma glucose level ≥ 5.1 mmol/L and/or 1 h glucose level ≥ 10.0 mmol/L and/or 2 h glucose level ≥ 8.5 mmol/L) [28, 29]. Nine NGT pregnant women were excluded from this study due to an insufficient stool sample weight. Four pregnant women with GDM were also excluded from our study: one who used drugs in vaginal suppositories, one who was obese, one who had hypothyroidism, and one who was positive for hepatitis B surface antigen. Ultimately, 33 stool samples were included in this study (15 from GDM and 18 from NGT pregnant women).

Patients were included in this study according to the following criteria: pregnant women with GDM and NGT aged 20–40 years of age; BMI ≤ 28 kg/m^2^ at first prenatal inspection; had not received any antibiotic treatments 1 month before sample collection; had not taken any probiotic medications 2 weeks before sample collection. Patients were excluded from the study according to the following criteria: history of diabetes, impaired glucose tolerance, hypertension, high cholesterol, thyroid disorders, asthma, fatty liver disease, inflammatory gastroenteritis, irritable bowel syndrome, cardiac, liver, or kidney diseases, psychiatric disorders, alcohol abuse, smoking, HIV, malignancy, illicit drug use (self-reported by the participant), or autoimmune or endocrine diseases prior to pregnancy. The study protocol was approved by the Ethics Committee of The Affiliated Changzhou No. 2. People’s Hospital of Nanjing Medical University (2018: KY304-01), and all patients provided written informed consent. This study was performed in accordance with the Declaration of Helsinki and Good Clinical Practice.

### Sample collection

To avoid surface and urine contamination, fresh stool samples (3–5 g) were collected in the morning with clean sterile spoons and placed in sterile and airtight tubes by the study participants. Each sample was suspended in sterile phosphate buffered saline (PBS; ~ 1 g/mL) and centrifuged at 2000 rpm for 5 min, and the residue was removed. Samples were centrifuged again at 3000 rpm for 8 min, and the supernatant was discarded before PBS and glycerin (1:1) were added to preserve the bacteria. All samples were stored at − 80 ℃ until transport. Stool collection was completed within 48 h of diagnosis.

### Bacterial DNA extraction from stool samples

Microbial community DNA was extracted using a MagPure Stool DNA KF kit B (Magen, Guangzhou, China) according to the manufacturer's instructions. DNA was quantified using a Qubit Fluorometer with a Qubit^®^ dsDNA BR assay kit (Invitrogen, Waltham, Massachusetts, USA), and the quality was checked by running an aliquot on a 1% agarose gel.

### 16S rRNA gene amplicon sequencing

The PCR system was configured using 30 ng genomic DNA samples of known quality and the corresponding fusion primers to set the PCR parameters for amplification. The V4 region of the bacterial 16S rRNA gene was amplified using the following degenerate PCR primers: 515F (5′-GTG CCA GCM GCC GCG GTA A-3′) and 806R (5′-GGA CTA CHV GGG TWT CTA AT-3′). The forward and reverse primers were tagged with Illumina adapter, pad, and linker sequences. PCR enrichment was performed in a 50 μL reaction volume containing 30 ng of template, fusion PCR primers, and PCR master mix. The cycling conditions were as follows: 95 °C for 3 min, 30 cycles of 95 °C for 45 s, 56 °C for 45 s, and 72 °C for 45 s, and a final extension at 72 °C for 10 min. PCR products were purified using Agencourt AMPure XP (Indianapolis, Indiana, USA) beads and eluted using an elution buffer (Omega Bio-tek, Norcross). Libraries were validated using an Agilent Technologies 2100 bioanalyzer (Agilent, Santa Clara, CA, USA) before being sequenced on an Illumina HiSeq 2500 platform (Illumina, San Diego, CA, USA) according to standard Illumina pipelines to generate 2 × 250 bp paired-end reads. Data were filtered by removing low-quality reads in each 25 bp window. Briefly, the entire sequence was removed if the final base was truncated from the window with an average quality of < 20 and if the read length after truncation was 75% lower than the original read length. Joint contamination reads, N-containing reads, and low complexity reads were also removed to obtain high-quality clean data. All bacterial 16S rRNA gene amplification, cloning, and sequencing of the PCR products were performed at BGI (Huada Gene Institute) Genomics (Shenzhen, China).

### QPCR verification of putative bacterial markers

To verify three potential bacterial markers (*R. bromii, C. colinum,* and *S. infantis*), we used qPCR with a common primer pair that can amplify most bacterial 16S rRNAs to normalize the levels of individual bacterial rRNA in each control and GDM sample to determine their detection sensitivity and specificity with primers selected from the primer database (GenBank genome and nucleotide). Generic primer pairs (Table 1) were used to normalize individual bacterial rDNA levels in the GDM and control samples. The qPCR results were further validated in the original subjects (*n* = 33).

**Table 1:** Primer sequences

Name	Taxonomy	Sequences (5ʹ–3ʹ)
*Ruminococcus bromii*	Genus	Forward: TTCAAGGACACCCACGAAGCA Reverse: AGTCGGCACAATAAACAAGACCAGT
*Clostridium colinum*	Genus	Forward: GACCTAACCGCAAGGAGGAG Reverse: CACCTTCCGATACGGCTACC
*Streptococcus infantis*	Genus	Forward: GTCTGTGATGAAGAAGCGGAATG Reverse: CTGGAGCCAAACTTGCGACTG
Common	All bacteria	Forward: AGAGCTACGAGCTGCCTGAC Reverse: AGCACTGTGTTGGCGTACAG

### Assessment of the biochemical specimens

Fasting venous blood samples (5 mL) were collected in a dry tube and centrifuged at 3000 rpm for 4 min, and the supernatant was collected. Biochemical indices were detected using a Cobas 8000 c702 automatic biochemical analyzer (Roche Diagnostics, Basel, Switzerland).

### Bioinformatics and statistical analysis

Raw reads were filtered to remove adaptors and low-quality and ambiguous bases before paired-end reads were added to tags using the Fast Length Adjustment of Short reads program (FLASH, v1.2.11) [30]. Tags were clustered into operational taxonomic units (OTUs) with a 97% cut-off value using UPARSE software (v7.0.1090) [31], and chimera sequences were compared with the Gold database using UCHIME (v4.2.40) [32]. Representative OTU sequences were taxonomically classified using Ribosomal Database Project Classifier (v2.2) with a minimum confidence threshold of 0.6 and were trained on the Greengenes database (v2.01305) in QIIME (v1.8.0) [33]. USEARCH_global [34] was used to compare all tags to OTUs to obtain OTU abundance statistics for each sample.

The number of tags for each taxonomic rank (species) or OTU in the samples was summarized using a profiling table or histogram in R (v3.1.1). Nonparametric statistics (Wilcoxon rank-sum test) were used to compare OTU abundance, bacterial communities, and alpha and beta diversity between the groups in Usearch. OTU alpha and beta diversity were estimated using MOTHUR (v1.31.2) [35] and QIIME (v1.8.0), respectively. Samples were clustered in QIIME (v1.8.0) using the unweighted pair group method with arithmetic mean (UPGMA).

Venn plots of OTUs or taxa were plotted using the “Venn diagram” package in R (v3.1.1). Partial least-squares discrimination analysis (PLS-DA) was performed using the mixOmics package in R (v3.2.1). Alpha diversity was analyzed using the Wilcox test in R (v3.2.1). LEfSe software (https://huttenhower.sph.harvard.edu/galaxy/) was used to determine markers with a linear discriminant analysis (LDA) of 2. Sequencing and PCR data were analyzed using unpaired *t* tests and Mann–Whitney rank-sum tests in GraphPad Prism. Categorical variables were analyzed using Chi-square and Wilcox tests in SPSS (v17.0). *P* values of < 0.05 were considered statistically significant. Linear regression analysis was used to assess the relationships between GM and GDM clinical parameters.

## Results

### Clinical characteristics of the participants

The clinical characteristics of the study participants are shown in Table 2. Statistically significant differences between the GDM and NGT groups were observed for the 1 h and 2 h OGTT glucose levels, which were significantly higher in the GDM group than in the NGT group (*P* < 0.001). Median weight during early pregnancy, BMI at the time of the OGTT visit, and weight gain from early pregnancy until the OGTT visit were also different between the two groups. The participants in the GDM group were slightly older than those in the NGT group. No differences were observed in median weight at the time of the OGTT visit, systolic blood pressure (SBP), diastolic blood pressure (DBP), fasting blood glucose (FBG), triglycerides, total cholesterol, gestational weeks, gravidity, or parity. Taken together, these results indicate that the significantly different clinical parameter between the GDM and NGT groups was primarily the OGTT blood glucose level.

**Table 2:** Clinical characteristics of the participants

Clinical parameters	GDM patients	NGT controls	*P* value
Number	15	18	–
Age, year	30.1 ± 3.5	26.1 ± 3.6	0.003
Height, cm^a^	160.0 (150, 172)	160.0 (157, 172)	0.334
Weight (early pregnancy), kg	63.9 ± 12.4	55.6 ± 6.6	0.031
BMI (early pregnancy), kg/m^2^	24.7 ± 4.1	21.1 ± 2.3	0.007
Weight (at OGTT), kg	70.4 ± 12.6	65.1 ± 7.5	0.156
BMI (at OGTT), kg/m^2^	27.2 ± 4.1	24.7 ± 2.6	0.043
Weight gain, kg^a^	6.0 (4.0, 9.5)	9.75 (2.5, 24.0)	0.014
SBP, mmHg	115.3 ± 9.3	115.7 ± 10.2	0.908
DBP, mmHg	68.1 ± 8.9	66.3 ± 7.3	0.515
FBG, mmol/L^a^	4.5 (4.1, 6.1)	4.4 (4.0, 5.1)	0.206
1 h OGTT glucose, mmol/L	10.1 ± 1.5	7.1 ± 1.2	< 0.001
2 h OGTT glucose, mmol/L	9.0 ± 1.5	6.3 ± 0.9	< 0.001
Triglycerides, mmol/L	2.9 ± 1.0	2.6 ± 0.9	0.476
Total cholesterol, mmol/L	6.2 ± 1.1	5.9 ± 1.1	0.396
Gestational weeks	26.4 ± 1.3	25.9 ± 1.7	0.364
Gravidity^a^	2 (1, 4)	1 (1, 3)	0.069
Parity^a^	0 (0, 1)	0 (0, 1)	0.058

Data presented as the mean ± SD

*BMI* body mass index, *OGTT* oral glucose tolerance test, *SBP* systolic blood pressure, *DBP* diastolic blood pressure, *FBG* fasting blood glucose. Weight gain indicates weight gain from early pregnancy until the OGTT visit. The 1 and 2 h OGTT indicate the 1 and 2 h blood glucose levels during the OGTT

^a^Data represented as the median value (minimum value, maximum value)

### Identification of OTU and GM composition in the GDM and NGT groups

**Fig. 1:**
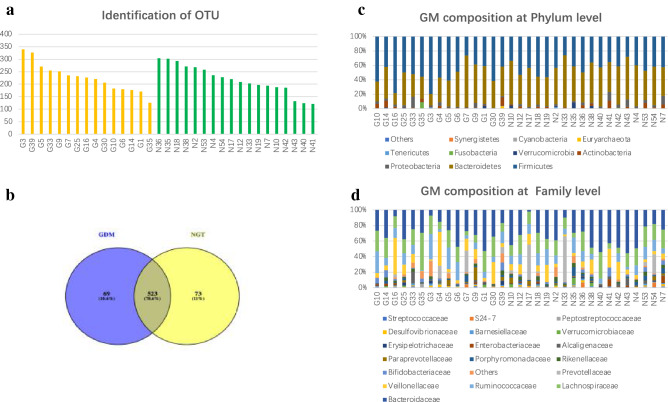
Identification of OTUs and GM composition in the GDM and NGT groups. **a** Identification of operational taxonomic units (OTUs). The yellow bar represents gestational diabetes mellitus (GDM) samples. The green bar represents control samples. **b** Venn diagram of OTU. Different colors represent different groups. The areas with overlapping circles of different colors represent the set of OTUs commonly present in the counterpart groups, and the single-layer zone represents the number of OTUs uniquely found in each group. **c** Histogram of GM composition at the phylum and **d** family levels in each sample between the GDM and NGT groups. Species whose abundance was less than 0.5% in all samples were merged into others. (G: gestational diabetes mellitus; N: normal glucose tolerance)

In total, 2,536,388 usable raw reads were obtained for all 33 samples, with an average of 76,860 reads per sample, and the read utilization ratio was 97.86%. All sequences were divided into 665 OTUs based on a 97% similarity level. The maximum identification of OTUs was 340, and the minimum was 121, with an average of 222. Up to 14 species were unique to each sample (Fig. 1a). Venn diagrams based on the OTU number distribution of samples from the two groups were created. Samples displayed high similarity between the two groups, with 523 shared OTUs; however, 69 OTUs were unique to the GDM group, and 73 were only found in the control group, indicating differences in species distribution (Fig. 1b).

Furthermore, the histogram visually displays the abundance of composition and the proportion of each sample species. At the phylum level, in total, 10 dominant phyla were identified across all the samples, with *Firmicutes, Bacteroidetes,* and *Proteobacteria* accounting for the majority of the total sequencing in both the GDM and NGT groups (Fig. 1c). At the class level, GM was mainly composed of *Bacteroidia*, *Clostridia,* and *Actinoteobacteria*. At the order level, GM was mainly composed of *Bacteroidales*, *Clostridiales,* and *Bifidobacteriales*. At the family level, GM was mainly composed of *Bacteroidaceae, Lachnospiraceae,* and *Ruminococcaceae*. At the genus level, GM was mainly composed of *Bacteroides, Prevotella,* and *Ruminococcus* in both the GDM and NGT groups (Fig. 1d). The GraPhlAn species composition showed the same results as the histograms (Fig. S1). In brief, these findings indicate that the quality of OTUs was good and that GM composition at multiple levels was relatively stable in the GDM and control groups.

### Identification of specific different species between the GDM and NGT groups

**Fig. 2:**
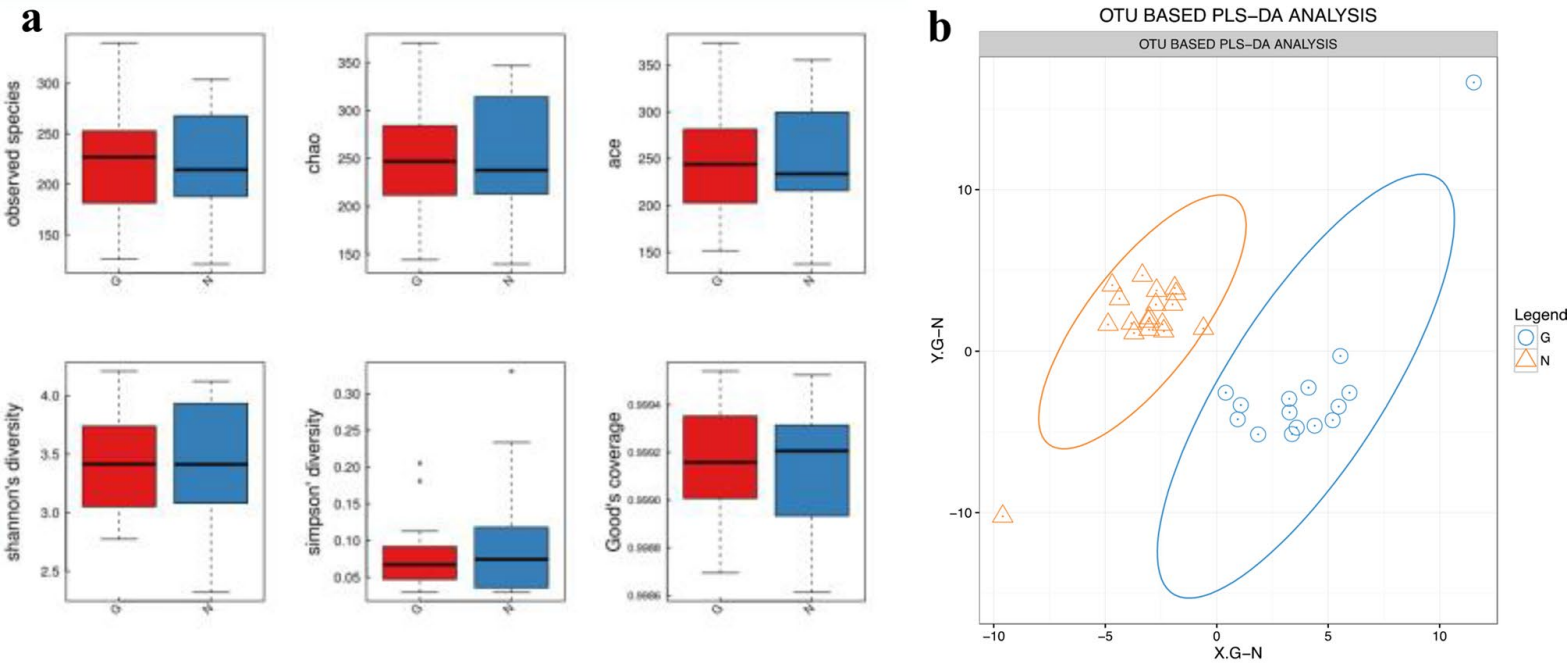
Alpha diversity and beta diversity between the GDM and control groups. **a** Alpha diversity analysis between gestational diabetes mellitus (GDM) and normal glucose tolerance (NGT) groups. The five lines from the bottom to the top are the minimum, first quartile, median, third quartile, and maximum, respectively. **b** OTU-based partial least-squares discrimination analysis (PLS-DA). The large ovals indicate good aggregation and significant differentiation between the GM structures of the GDM and NGT groups (G: GDM; N: NGT)

To determine whether there was a significant difference in alpha diversity and beta diversity in GM between the GDM and NGT groups, we used six indices (observed species, Chao, ACE, Shannon’s diversity, Simpson’s diversity, and Good’s coverage) to indicate alpha diversity and partial least-squares discrimination analysis (PLS-DA) to indicate beta diversity. There was no significant difference in alpha diversity (Fig. 2a). However, the PLS-DA of the OTUs in each group indicated good aggregation and significant differentiation of GM structures between the GDM and the control groups (Fig. 2b).

**Fig. 3:**
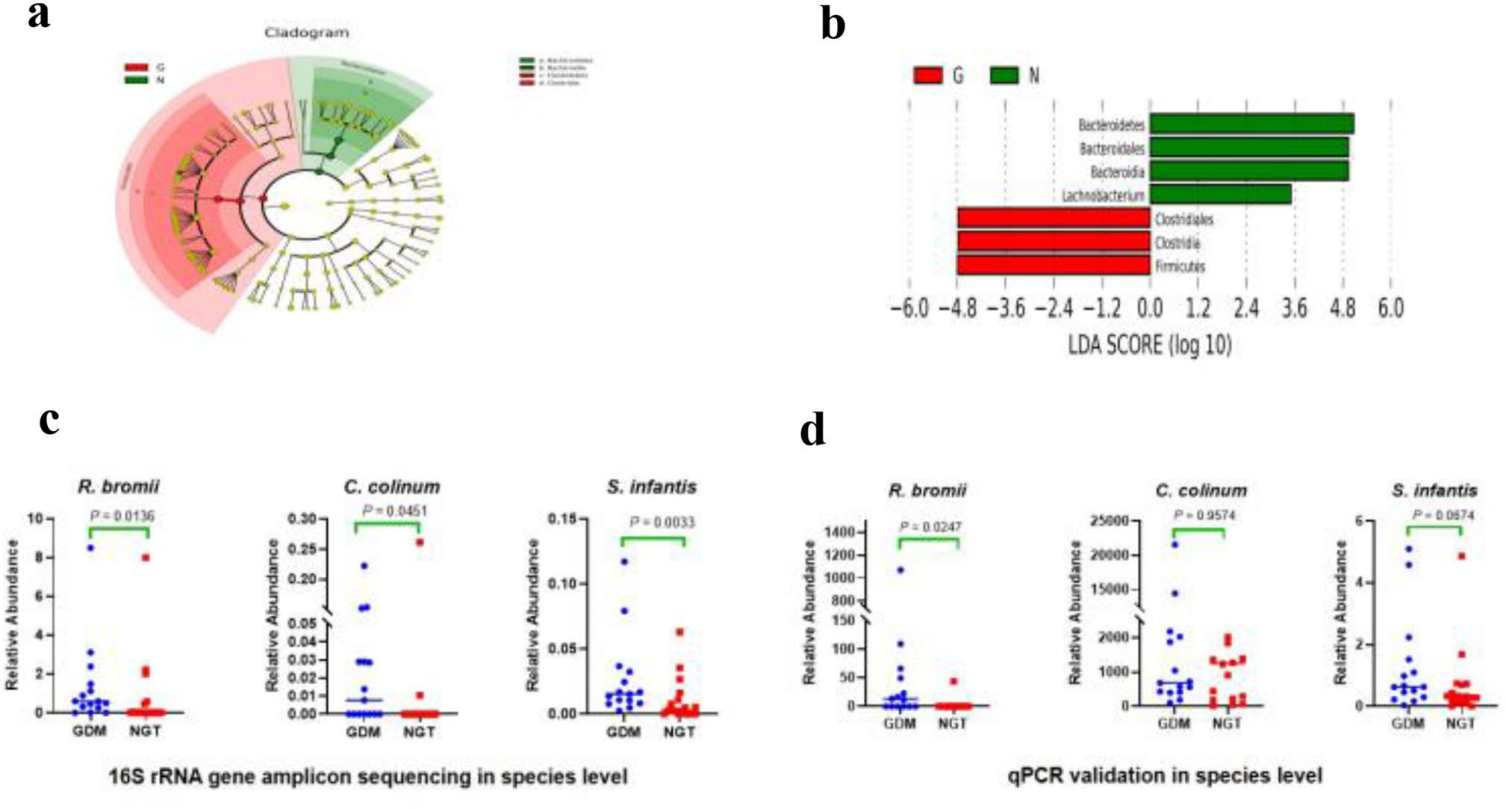
Identification of specific different species between the GDM and control groups. **a** Cladogram (LEfSe DA clustering tree); **b** linear discriminant analysis (LDA) scores from LEfSe (LDA effect size) were used on the genus-level OTU tables to determine the taxa that best characterized each biological class. Red: gestational diabetes mellitus (GDM); green: normal glucose tolerance (NGT); **c** relative abundance at the species level in sequencing between the GDM and the control groups: *R. bromii, C. colinum*, and *S. infantis* were significantly increased in the GDM data*;* **d** relative abundance at the species level in qPCR between the GDM and the control groups: *R. bromii* and *S. infantis* were significantly increased in the GDM data*. P* values are indicated on each graph; *P* values of *S. infantis* = 0.018 after adjusting for BMI1 + BMI2 (linear regression)

LEfSe analysis discovered a significant difference between the GDM and NGT groups through biometric and statistically significant differences. Linear discriminant analysis (LDA) can obtain reliable results through dimensionality reduction technology. Comparing stool samples of the GDM group with the control group, LEfSe analysis (Fig. 3a) and the LDA score (Fig. 3b) both revealed that *Clostridiales*, *Clostridia*, and *Firmicutes* were significantly associated with the GDM samples, while *Bacteroidetes, Bacteroidia*, and *Bacteroidales* (*Lachnobacterium specific* in the LDA score) were related to the control samples. Moreover, based on PLS-DA analysis and the diff_wilcoxon-test of sequencing, at the species level, a great difference in *R. bromii, C. colinum*, and *S. infantis* was found between the GDM and NGT groups (*P* < 0.05; Fig. 3c; Table S1).

In short, beta diversity identified the specific differences in the species of *R. bromii, C. colinum*, and *S. infantis* between the GDM and NGT groups.

### qPCR validation of putative bacterial biomarkers

To verify the sequencing analysis findings at the species level, we quantified the levels of the identified species in the same subjects with GDM and NGT by qPCR. The results show that samples from the GDM group contained a higher abundance of *R. bromii* (*P* < 0.05) and *S. infantis* (*P* < 0.1) compared to the NGT group, consistent with the sequencing results (Fig. 3d). Therefore, these qPCR data validated that *R. bromii* and *S. infantis* as differential biomarkers between the GDM and NGT groups was highly credible.

### Correlation between the identified species and the clinical characteristics

**Fig. 4:**
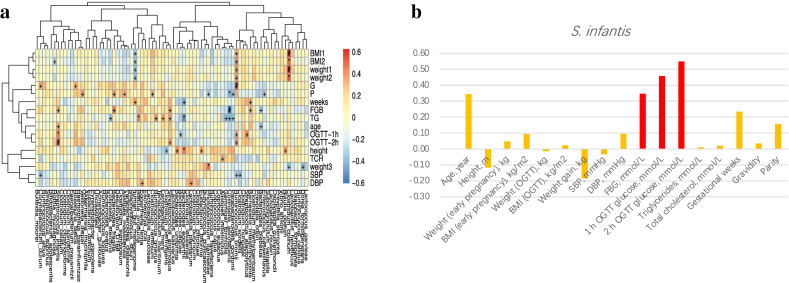
Correlation between the identified species and the clinical characteristics.** a** Heatmap represents the correlation coefficients between the identified species and the clinical characteristics of gestational diabetes (GDM). In the box or bar, # indicates a *P* value < 0.001, * indicates when 0.001 < *P* value < 0.01 and + indicates when 0.01 < *P* value < 0.05; **b** histogram representing the correlation coefficients between *S. infantis* and the clinical characteristics of GDM. Red represents a positive correlation (*P* < 0.001)

From the heatmap that showed the correlation between the identified species and the clinical parameters (Fig. 4a; Table S2), blood glucose and body weight both appeared to be closely related to the identified species. After adjusting for body weight (BMI1 and BMI2), *S. infantis* still showed a significant correlation with the GDM clinical characteristics (*P* < 0.05; Table S3). After adjusting for age, *S. infantis* did not show a significant correlation with the GDM clinical characteristics (*P* > 0.05; Table S3). This may be due to an insufficient sample size or an insufficient effect size of *S. infantis*. This study found *S. infantis* had a strong positive correlation with FBG (*P* < 0.05) and the 1 h and 2 h OGTT glucose levels (*P* < 0.01 and < 0.001, respectively) (Fig. 4b). In conclusion, *S. infantis* has a close relationship with blood glucose, which is a risk factor for GDM.

## Discussion

The human microecological system contains a large number of microorganisms referred to as the gut microbiota (GM), which generate more than 35% of the enzymes required for human metabolism. In addition, the GM plays key roles in many of the main systems of the human body, including the immune, nervous, endocrine, digestive, respiratory, and circulatory systems [36]. Microbial dysbiosis in the human GM may be an important environmental risk factor for abnormal host metabolism [24]. During normal pregnancy, a low grade of inflammation develops [37]. The stools of the third trimester showed the strongest signs of inflammation and energy loss [18]. GDM is a pro-inflammatory state [37]. An imbalance in pro- and anti-inflammatory bacterial species has been proposed to trigger low-grade inflammation and insulin resistance in humans [27]. Ferrocino et al. found a strong inverse relationship between *Faecalibacterium* abundance and fasting glucose values [38], supporting the well-known association between inflammation and dysmetabolism [27].

In our study, LEfSe analysis and the LDA score both revealed that *Clostridiales*, *Clostridia*, and *Firmicutes* were significantly associated with GDM samples, while *Bacteroidetes*, *Bacteroidia*, and *Bacteroidales* were related to control samples, which is generally consistent with the results of previous studies [18, 38, 39]. Although there was no difference in the alpha diversity of GM, the beta diversity was significantly different at the species level (*R. bromii*, *C. colinum*, and *S. infantis*) between the GDM and NGT groups, consistent with the qPCR results, which indicated higher levels of *R. bromii* and *S. infantis* in the GDM group. These findings suggest that GM is different between the GDM and NGT groups and plays an important role in GDM development; however, the specific mechanisms require further investigation.

*R. bromii* belongs to the phylum *Firmicutes* and the genus *Ruminococcus*, and previous studies have shown that low *Proteobacteria* and high *Ruminococcus* abundance are associated with a healthy GM [40], which is important for a symbiotic relationship with the host. *Firmicutes* have also been shown to correlate negatively with resting energy expenditure and positively with fat mass percentage [41]. Indeed, a crossover clinical trial observed that a 20% increase in *Firmicutes* abundance is associated with an energy harvest increase of 150 kcal [42]. *R. bromii* has also been shown to be an important taxon involved in the degradation of resistant starch into butyric acid in diabetes treatments; however, no studies have yet reported the relationship between *R. bromii* and GDM. In this study, statistical analysis of the relationship between the identified species and the clinical parameters revealed that *R. bromii* had a strong positive correlation with gravidity, indicating that *R. bromii* may be related to placental hormones such as progesterone and estrogen or to insulin secretion. In addition, we found that *R. bromii* had a close positive relationship with the glucose level of the OGTT and body weight. Since previous studies [43] have suggested that *R. bromii* may play a role in promoting weight gain and blood glucose levels in women with GDM, our sequencing and qPCR results indicate that *R. bromii* is a mark of GDM and obesity.

*C. colinum* is a close relative of *C. piliforme*, which causes ulcerative enteritis in young game birds, chickens, turkeys, and occasionally other avian species. Very little is known about the pathogenesis of this infection, since the *C. colinum* genome has not yet been characterized; the basis of its remarkable virulence is unknown, and no reports of *C. colinum* infection in nonavian species have been published [44]. In this study, we found a significant relationship between *C. colinum* and weight, weight gain, and BMI during pregnancy; we also found a strong association between *C. colinum* and BMI when adjusted for age, FGB, and 1 h and 2 h OGTT levels (*P* < 0.05; Table S3). Thus, *C. colinum* may be a mark of obesity. These findings are consistent with previous studies that have reported positive associations between *Clostridium* and obesity [31, 45–48]. *C. colinum* belongs to the phylum *Firmicutes*, which contains many butyrate-producing species that may increase energy harvest in obese individuals along with increased acetate synthesis [49, 50]. The mechanisms by which the microbiome and particular species affect obesity remain unclear; therefore, future studies should investigate *C. colinum* further.

*S. infantis* belongs to the genus *Streptococcus*, which includes more than 50 species. Few previous studies have reported the characteristics of *S. infantis*, likely due to their very low abundance in GM, with glutamate dehydrogenase PCR failing to amplify *S. infantis* sequences [51]. Increased age and weight are known to be associated with GDM [18, 52], but in our study, we did not observe a significant association between age and the identified species. There was no obvious association between *S. infantis* and GDM clinical parameters when linear regression analysis was used to adjust for age. This may be due to an insufficient sample size or an insufficient effect size of *S. infantis*. Conversely, body weight may have been a highly important risk factor closely related to GDM in our study. Therefore, we used linear regression analysis to adjust the factor of body weight and found that *S. infantis* still plays an important role in pregnant women with GDM.

Recently, Kilian et al. demonstrated that *S. infantis* strains are an important constituent of the oral commensal microbiota. *S. infantis* forms distinct bacterial populations that are less coherent than traditionally expected for bacterial species. The proportion of oral *S. infantis* is negatively correlated with the elimination of bacteria [53]. *S. infantis* can affect transmembrane barrier transport by changing the expression of capsular polysaccharides or the expression of related genes, thereby affecting the body's energy metabolism, especially the carbohydrate transport system [53]. It has been reported that *S. infantis* is associated with periodontitis through submucosal biofilms [54] and Behcet’s disease through T-cell aberration by GM metabolite alteration [55]. As an abundant colonizer in the upper respiratory tract and oral cavity, *S. infantis* was also demonstrated in a recent study in which fecal samples with a signature of high SARS-CoV-2 infectivity had a higher abundance of *S. infantis* and a higher functional capacity for nucleotide de novo biosynthesis, amino acid biosynthesis, and glycolysis [56].

The GM is associated with metabolic impairment and inflammation [18]. The GM affects maternal insulin function and induces GDM through several mechanisms: (i) the GM affects the maternal nervous system through the gut–brain axis, reduces insulin receptor sensitivity, increases insulin resistance, and induces GDM [57]; (ii) the GM activates the Nod/Rip2 signaling pathway in β-cells, affects intestinal epithelial cells to release Nod ligands, and leads to insufficient β-cell function and GDM [58]; (iii) the GM connects receptors of GPR-41 and GRP-43 on enteroendocrine L cells through various mediators (such as short-chain fatty acids and intestinal hormones) that act on target organs, stimulate the release of GLP-1 and PYY, regulate energy intake, cause energy metabolism disorders, and induce GDM [59]; (iv) the GM affects the immune system of pregnant women. When the butyrate produced by the GM decreases, the intestinal mucosa is damaged, permeability increases or immunity is imbalanced, immune cells infiltrate, and pro-inflammatory cytokines (INF-a, IL-6) increase, activating the inflammatory signaling pathway and leading to insulin resistance and GDM [60–63].

Our study revealed a clearly significant difference in *S. infantis* between the GDM and NGT groups and identified a strong positive relationship between *S. infantis* and OGTT blood glucose levels (particularly the OGTT 2 h glucose level, *P* < 0.0001). Taken together, we are not confident whether we can hypothesize that *S. infantis* is involved in the mechanism of inflammation and metabolic imbalance, causing local inflammation in the GM and activating systemic inflammation, leading many inflammatory cytokines to be released into target organs where insulin acts, inhibiting the activity of proteins related to the insulin signaling pathway, causing β-cell damage or necrosis, and ultimately inducing insulin resistance and GDM disease. In other words, *S. infantis* may serve as a mark for GDM, and its role in the causality and biological relevance must be further verified in animal model studies.

The notable strength of this study is the first report that *S. infantis* has a very strong positive association with blood glucose levels, thus providing a new research avenue for future studies. However, this study also has several limitations. First, the sample sizes used for sequencing analysis were not large enough to allow for strong conclusions to be drawn. Additional studies with larger sample sizes are therefore warranted in the future to validate the findings of the current study. Second, body weight, which acts as a confounding factor, was not completely excluded from our study. Although one patient in the GDM group had a BMI > 30, the inclusion of this patient did not affect our results, with similar results observed when the subject was excluded from analysis or after adjusting for BMI. More standard subjects will be required in future studies to obtain more accurate and stronger proof. Finally, gold standard tests for measuring insulin resistance and secretion were not performed in this study, and no information was obtained regarding pancreatic β-cell function in the patients with GDM during pregnancy. Therefore, to provide a more comprehensive overview of each patient to draw more definitive conclusions, it will be important to include these tests in future studies.

## Conclusion

This study identified three abnormally expressed intestinal bacteria (*R. bromii*, *C. colinum*, and *S. infantis*) in women with GDM. Further correlation analysis revealed that *S. infantis* has a strong relationship with blood glucose levels and may serve as a disease risk factor for GDM. Thus, if these findings are confirmed by further studies in a larger sample, the development of strategies to modulate the GM might provide a new avenue for treating GDM.

## Supplementary Information

Below is the link to the electronic supplementary material.

Supplementary file1 (PDF 306 kb)

## Data Availability

The sequence and metadata have been deposited in GreenGene [accession number: F19FTSECWLJ1462_HOMIsvM.1]. All data used for the analysis in this article are available on request from the authors.

## Abbreviations

GDM: Gestational diabetes mellitus
NGT: Normal glucose tolerance
GM: Gut microbiota
T2DM: Type 2 diabetes mellitus
OGTT: Oral glucose tolerance test
FBG: Fasting blood glucose
OTU: Operational taxonomic unit
PLS-DA: Partial least-squares discrimination analysis
LDA: Linear discriminant analysis
PBS: Phosphate-buffered saline
qPCR: Quantitative polymerase chain reaction
BMI: Body mass index
SBP: Systolic blood pressure
DBP: Diastolic blood pressure
DNA: Deoxyribose nucleic acid
rRNA: Ribosomal RNA
g: Gram(s)
h: Hour(s)
ml: Milliliter(s)
°C: Degree centigrade

## References

[1] Hanson MAGP (2015). Developmental origins of health and disease—global public health implications. Best Pract Res Clin Obstet Gynaecol.

[2] Longmore DKBE, Lee I (2019). Maternal body mass index, excess gestational weight gain, and diabetes are positively associated with neonatal adiposity in the Pregnancy and Neonatal Diabetes Outcomes in Remote Australia (PANDORA) study. Pediatr Obes.

[3] Carroll XLX, Zhang W (2018). Socioeconomic, environmental and lifestyle factors associated with gestational diabetes mellitus: a matched case-control study in Beijing, China. Sci Rep.

[4] Chiefari EAB, Foti D (2017). Gestational diabetes mellitus: an updated overview. J Endocrinol Invest.

[5] Wei JGJ, Cheng J (2014). Gestational diabetes mellitus and impaired glucose tolerance pregnant women. Pakistan J Med Sci.

[6] Metzger BEBT, Coustan DR (2007). Summary and recommendations of the fifth international workshop-conference on gestational diabetes mellitus. Diabetes Care.

[7] Wei JLX, Gao J (2015). Insulin secretion and tolerance of women with different gestational glucose regulation one year postpartum. Int J Clin Exp Med.

[8] Lauenborg JHT, Jensen DM (2004). Increasing incidence of diabetes after gestational diabetes: a long-term follow-up in a Danish population. Diabetes Care.

[9] Damm PHOA, Kelstrup L (2016). Gestational diabetes mellitus and long-term consequences for mother and offspring: a view from Denmark. Diabetologia.

[10] Agha-Jaffar RON, Johnston D (2016). Gestational diabetes mellitus: does an effective prevention strategy exist?. Endocrinology.

[11] Eyupoglu NDCGE, Acikgoz A (2019). Circulating gut microbiota metabolite trimethylamine N-oxide and oral contraceptive use in polycystic ovary syndrome. Clin Endocrinol (Oxford).

[12] Zhou LXX, Zhang Q (2019). Gut microbiota might be a crucial factor in deciphering the metabolic benefits of perinatal genistein consumption in dams and adult female offspring. Food Funct.

[13] Jiao NBS, Nugent CA (2018). Gut microbiome may contribute to insulin resistance and systemic inflammation in obese rodents: a meta-analysis. Physiol Genom.

[14] Simpson SSL, Bowe J (2018). Placental peptides regulating islet adaptation to pregnancy: clinical potential in gestational diabetes mellitus. Curr Opin Pharmacol.

[15] Kalra SGY, Kumar A (2016). Prevention of gestational diabetes mellitus (GDM). J Pakistan Med Assoc.

[16] Kemp PFAJ (2004). Bacterial diversity in aquatic and other environments: what 16S rDNA libraries can tell us. FEMS Microbiol Ecol.

[17] DiGiulio DBCB, McMurdie PJ (2015). Temporal and spatial variation of the human microbiota during pregnancy. PNAS.

[18] Koren OGJ, Cullender T (2012). Host remodeling of the gut microbiome and metabolic changes during pregnancy. Cell (Cambridge).

[19] Kuang YLJ, Li S (2017). Connections between the human gut microbiome and gestational diabetes mellitus. Gigascience.

[20] Crusell MKWHT, Nielsen T (2018). Gestational diabetes is associated with change in the gut microbiota composition in third trimester of pregnancy and postpartum. Microbiome.

[21] Kirwan JPVA, Jing M (2004). Reversal of insulin resistance postpartum is linked to enhanced skeletal muscle insulin signaling. J Clin Endocrinol Metab.

[22] Hold GLPS, Russell VJ (2002). Assessment of microbial diversity in human colonic samples by 16S rDNA sequence analysis. FEMS Microbiol Ecol.

[23] Hod MKA, Sacks DA (2015). The International Federation of Gynecology and Obstetrics (FIGO) Initiative on gestational diabetes mellitus: a pragmatic guide for diagnosis, management, and care. Int J Gynecol Obstetr.

[24] Karlsson FTV, Nielsen J (2013). Assessing the human gut microbiota in metabolic diseases. Diabetes (New York, NY).

[25] Jones MLMC, Ganopolsky JG (2014). The human microbiome and bile acid metabolism: dysbiosis, dysmetabolism, disease and intervention. Expert Opin Biol Ther.

[26] Fang SER (2013). Microbiology: wealth management in the gut. Nature (London).

[27] Le Chatelier ENT, Qin J (2013). Richness of human gut microbiome correlates with metabolic markers. Nature (London).

[28] Mago TSS (2011). FLASH: fast length adjustment of short reads to improve genome assemblies. Bioinformatics.

[29] Rc E (2013). UPARSE: highly accurate OTU sequences from microbial amplicon reads. Nat Methods.

[30] Edgar RCHB, Clemente JC (2011). UCHIME improves sensitivity and speed of chimera detection. Bioinformatics.

[31] Caporaso JGKJ, Stombaugh J (2010). QIIME allows analysis of high-throughput community sequencing data. Nat Methods.

[32] Rc E (2010). Search and clustering orders of magnitude faster than BLAST. Bioinformatics (Oxford, England).

[33] Schloss PDWS, Ryabin T (2009). Introducing mothur: open-source, platform-independent, community-supported software for describing and comparing microbial communities. Appl Environ Microbiol.

[34] Johns ECDF, Norman JE (2018). Gestational diabetes mellitus: mechanisms, treatment, and complications. Trends Endocrinol Metab.

[35] Olsson LMPC, Tremaroli V (2019). Gut microbiota of obese subjects with Prader-Willi syndrome is linked to metabolic health. Gut.

[36] Huang LWT, Wu Q (2019). Analysis of microbiota in elderly patients with Acute Cerebral Infarction. PeerJ (San Francisco, CA).

[37] Röytiö HMK, Vahlberg T (2018). Dietary intake of fat and fibre according to reference values relates to higher gut microbiota richness in overweight pregnant women—CORRIGENDUM. Br J Nutr.

[38] Ferrocino IPV, Gambino R (2018). Changes in the gut microbiota composition during pregnancy in patients with gestational diabetes mellitus (GDM). Sci Rep.

[39] Karlsson FHTV, Nookaew I (2013). Gut metagenome in European women with normal, impaired and diabetic glucose control. Nature (London).

[40] Hollister EBGC, Versalovic J (2014). Compositional and functional features of the gastrointestinal microbiome and their effects on human health. Gastroenterology (New York, NY 1943).

[41] Kocełak PZGA, Zahorska-Markiewicz B (2013). Resting energy expenditure and gut microbiota in obese and normal weight subjects. Eur Rev Med Pharmacol Sci.

[42] Jumpertz RLD, Turnbaugh PJ (2011). Energy-balance studies reveal associations between gut microbes, caloric load, and nutrient absorption in humans. Am J Clin Nutr.

[43] Jandhyala SMMA, Deepika G (2017). Altered intestinal microbiota in patients with chronic pancreatitis: implications in diabetes and metabolic abnormalities. Sci Rep.

[44] Cooper KKSJ, Uzal FA (2013). Diagnosing clostridial enteric disease in poultry. J Vet Diagn Invest.

[45] Collado MCIE, Laitinen K (2008). Distinct composition of gut microbiota during pregnancy in overweight and normal-weight women. Am J Clin Nutr.

[46] Collado MCIE, Laitinen K (2010). Effect of mother’s weight on infant’s microbiota acquisition, composition, and activity during early infancy: a prospective follow-up study initiated in early pregnancy. Am J Clin Nutr.

[47] Damms-Machado AMS, Schollenberger AE (2015). Effects of surgical and dietary weight loss therapy for obesity on gut microbiota composition and nutrient absorption. Biomed Res Int.

[48] Remely MTI, Hippe B (2015). Gut microbiota composition correlates with changes in body fat content due to weight loss. Benef Microb.

[49] Gordon JILR, Klein S (2006). Microbial ecology Human gut microbes associated with obesity. Nature (London).

[50] Schwiertz ATD, Schäfer K (2010). Microbiota and SCFA in lean and overweight healthy subjects. Obesity (Silver Spring, Md).

[51] Hoshino TFT, Kilian M (2005). Use of phylogenetic and phenotypic analyses to identify nonhemolytic streptococci isolated from bacteremic patients. J Clin Microbiol.

[52] Solomon CGWW, Carey VJ (1997). A prospective study of pregravid determinants of gestational diabetes mellitus. JAMA.

[53] Kilian M, Tettelin H (2019) Identification of virulence-associated properties by comparative genome analysis of *Streptococcus**pneumoniae*, *S. pseudopneumoniae*, *S. mitis*, three *S. oralis* Subspecies, and *S. infantis*. mBio 10(5) 10.1128/mBio.01985-19 PMC6722419 31481387

[54] Yu XCY, Zhuang L (2019). Intra-oral single-site comparisons of periodontal and peri-implant microbiota in health and disease. Clin Oral Implants Res.

[55] Shimizu JKT, Takada E (2019). Relative abundance of Megamonas hypermegale and Butyrivibrio species decreased in the intestine and its possible association with the T cell aberration by metabolite alteration in patients with Behcet's disease (210 characters). Clin Rheumatol.

[56] Zuo T, Liu Q, Zhang F, Lui GC, Tso EY, Yeoh YK (2021). Depicting SARS-CoV-2 faecal viral activity in association with gut microbiota composition in patients with COVID-19. Gut.

[57] Osadchiy VMC, Mayer EA (2019). The gut-brain axis and the microbiome: mechanisms and clinical implications. Clin Gastroenterol Hepatol.

[58] Zhang QPY, Zeng B (2019). Intestinal lysozyme liberates Nod1 ligands from microbes to direct insulin trafficking in pancreatic beta cells. Cell Res.

[59] Cani PDVHM, Lefort C (2019). Microbial regulation of organismal energy homeostasis. Nat Metab.

[60] Mehta NNMF, Anderson PD (2010). Experimental endotoxemia induces adipose inflammation and insulin resistance in humans. Diabetes (New York, NY).

[61] Wolf MSJ, Shah A (2004). Inflammation and glucose intolerance: a prospective study of gestational diabetes mellitus. Diabetes Care.

[62] TE Davis-Richardson AG (2015). A model for the role of gut bacteria in the development of autoimmunity for type 1 diabetes. Diabetologia.

[63] Se S (2006). Inflammation and insulin resistance. J Clin Investig.

