# Supplementary material for: 16S rRNA gene amplicon sequencing of gut microbiota in gestational diabetes mellitus and their correlation with disease risk factors
Source: J Endocrinol Invest. 2021 Jul 24;45(2):279–89. doi: 10.1007/s40618-021-01595-4 (PMC8308075; doi:10.1007/s40618-021-01595-4)
Supplement: Supplementary file 1 — Supplementary file1 (PDF 306 kb) [file 40618_2021_1595_MOESM1_ESM.pdf]

Supplementary Figure and Tables

Fig. S1

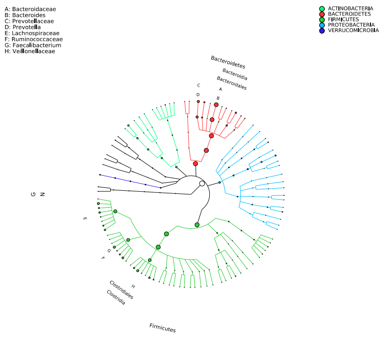

Fig. S1 GraPhlAn species composition

From outside to inside, each circle represents a level, phylum, class, order, family, genus, and species, respectively. The circle outside represents the heatmap of abundance. Different colors represent the different phyla. The shade of color represents the abundance of the species. The larger the node is, the larger the abundance of the species.

Table S1 Identification of specific different between the GDM and the NGT groups

Species bacterial	Mean(G)	SD(G)	Mean(N)	SD(N)	*P* value
R. bromii	1.35	2.17	0.74	1.93	0.014
C. colinum	0.04	0.07	0.02	0.06	0.045
S. infantis	0.03	0.03	0.01	0.02	0.003

Note: *P* value is for sequencing (diff_Wilcoxon-test).

G indicates GDM patients; N indicates NGT controls

Table S2 Correlation between the identified species and the clinical characteristics

Clinical parameters	*R. bromii*		*C. colinum*		*S. infantis*	
	r	*P*	*r*	*P*	*r*	*P*
Age, year	0.16	0.39	0.2	0.28	0.34	0.05
Height, m	0.05	0.78	0.16	0.39	-0.12	0.51
Weight (early pregnancy), kg	0.42	0.02	0.62	<0.001	0.049	0.79
BMI (early pregnancy), kg/m^2	0.37	0.03	0.63	<0.001	0.09	0.6
Weight (OGTT), kg	0.41	0.02	0.53	0.002	-0.01	0.94
BMI (OGTT), kg/m^2	0.42	0.02	0.45	0.003	0.02	0.9
Weight gain, kg	-0.19	0.3	-0.37	0.03	-0.18	0.3
SBP, mmHg	-0.35	0.048	-0.07	0.7	-0.03	0.85
DBP, mmHg	-0.14	0.42	0.16	0.38	0.1	0.6
FBG, mmol/L	0.09	0.63	0.11	0.53	0.35	0.048
1 h OGTT glucose, mmol/L	0.37	0.04	0.23	0.2	0.46	0.008
2 h OGTT glucose, mmol/L	0.41	0.02	0.22	0.22	0.55	<0.001
Triglycerides, mmol/L	0.1	0.58	0.14	0.44	0.01	0.95
Total cholesterol, mmol/L	-0.34	0.05	0.05	0.78	0.02	0.91
Gestational weeks	0.18	0.33	0.06	0.76	0.23	0.19
Gravidity	0.57	<0.001	0.0006	0.997	0.04	0.85
Parity	0.33	0.06	0.01	0.94	0.16	0.39

Data presented as the mean ± SD

[a] Data represented as the median value (minimum value, maximum value)

BMI: body mass index, OGTT: oral glucose tolerance test, SBP: systolic blood pressure, DBP: diastolic blood pressure, FBG: fasting blood glucose. Weight gain indicates weight gain from early pregnancy until the OGTT visit. The 1 and 2 h OGTT indicate the 1 and 2 h blood glucose levels during OGTT.

Table S3 Unadjusted and adjusted association of GDM with the identified species

Bacterial Species	GDM ß Coefficient (95%CI)									
	Unadjusted	*P* value	Model 1	*P* value 1	Model 2	*P* value 2	Model 3	*P* value 3	Model 4	*P* value 4
R. bromii	0.61(-0.79,2.01)	0.402	0.82(-0.77,2.42)	0.320	0.15（-1.47,1.76)	0.859	0.55(-1.31,2.42)	0.567	-1.72(-3.91,0.48)	0.137
C. colinum	0.02(-0.02,0.07)	0.321	-0.02(-0.07,0.03)	0.424	0.04(-0.02,0.09)	0.192	-0.01(-0.06,0.05)	0.788	0.09(-0.11,0.36)	0.03
S. infantis	0.02(-0.00,0.03)	0.062	0.03(0.01, 0.05)	0.018	0.08(-0.01,0.03)	0.431	0.01(-0.01,0.04)	0.201	0.007(-0.02,0.03)	0.645

Note: Model 1 adjusted for BMI1 and BMI2

Model 2 adjusted for age

Model 3 adjusted for Model 1 and Model 2

Model 4 adjusted for age, FGB, and OGTT 1 h and 2 h levels
